# Supplementary figures and images for: The Gene Expression Profile of Uropathogenic Escherichia coli in Women with Uncomplicated Urinary Tract Infections Is Recapitulated in the Mouse Model
Source: mBio. 2020 Aug 11;11(4):e01412-20. doi: 10.1128/mBio.01412-20 (PMC7439467; doi:10.1128/mBio.01412-20)

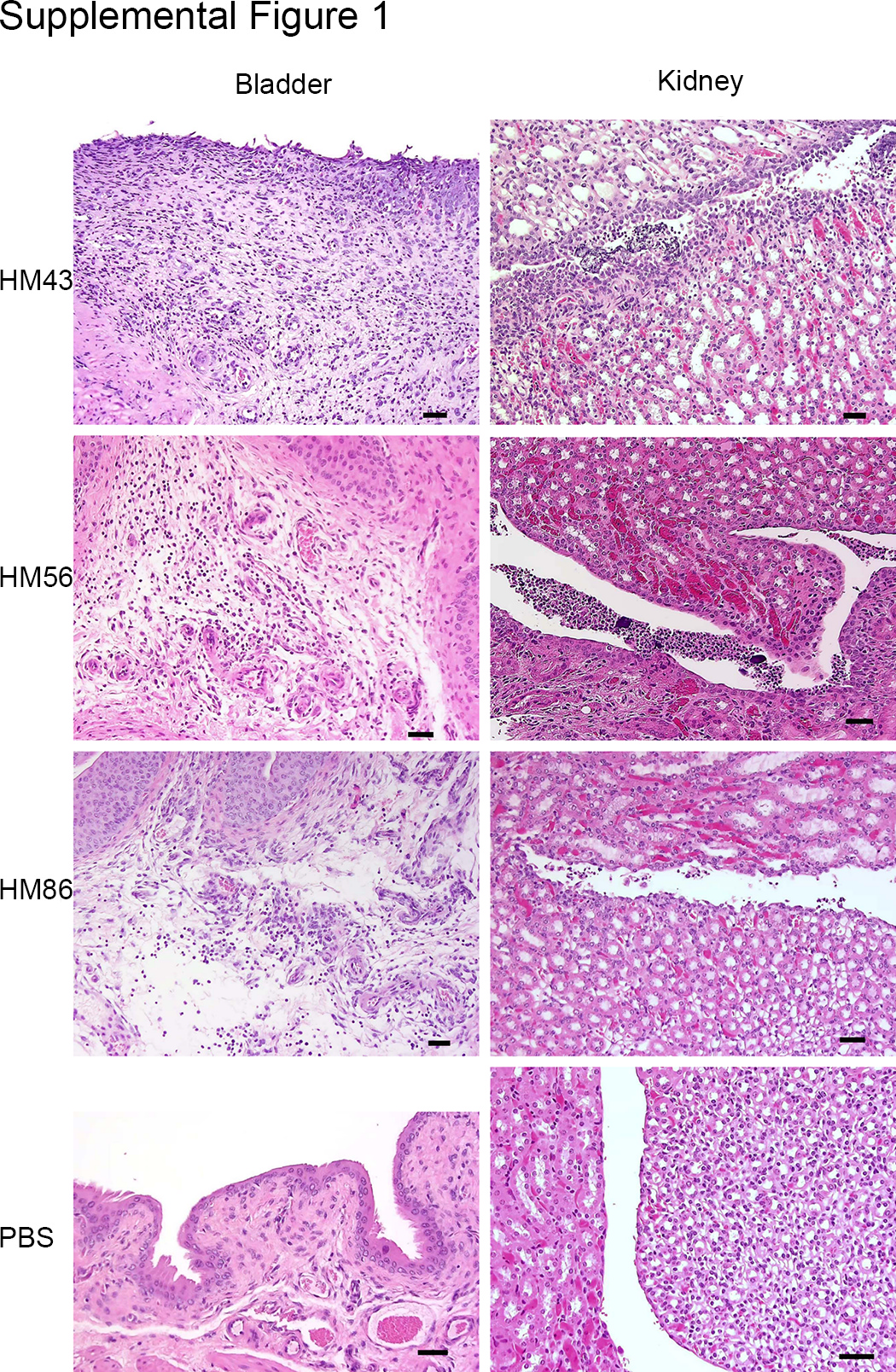

Supplement: FIG S1 [file mBio.01412-20-sf001.tif]

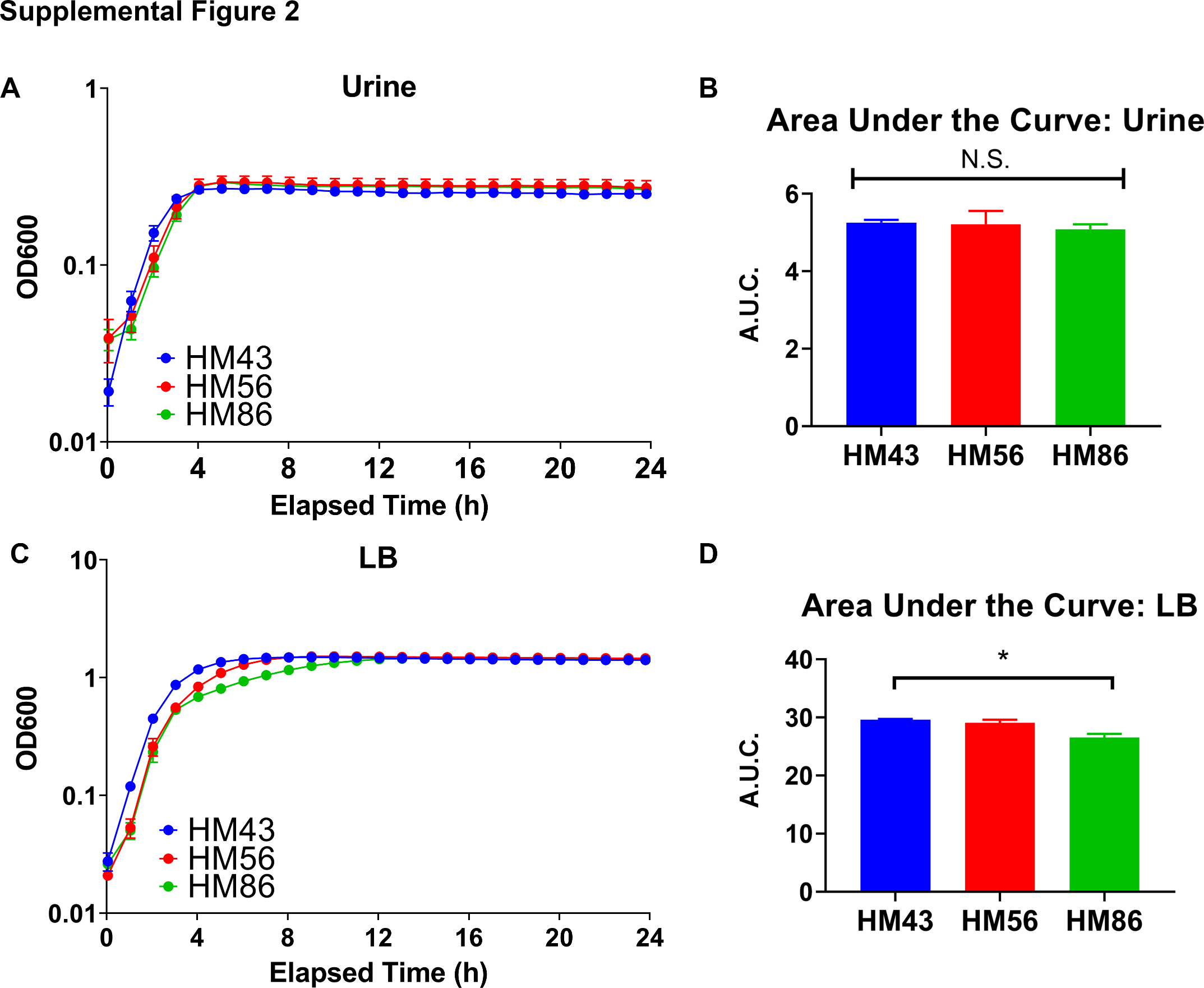

Supplement: FIG S2 [file mBio.01412-20-sf002.tif]

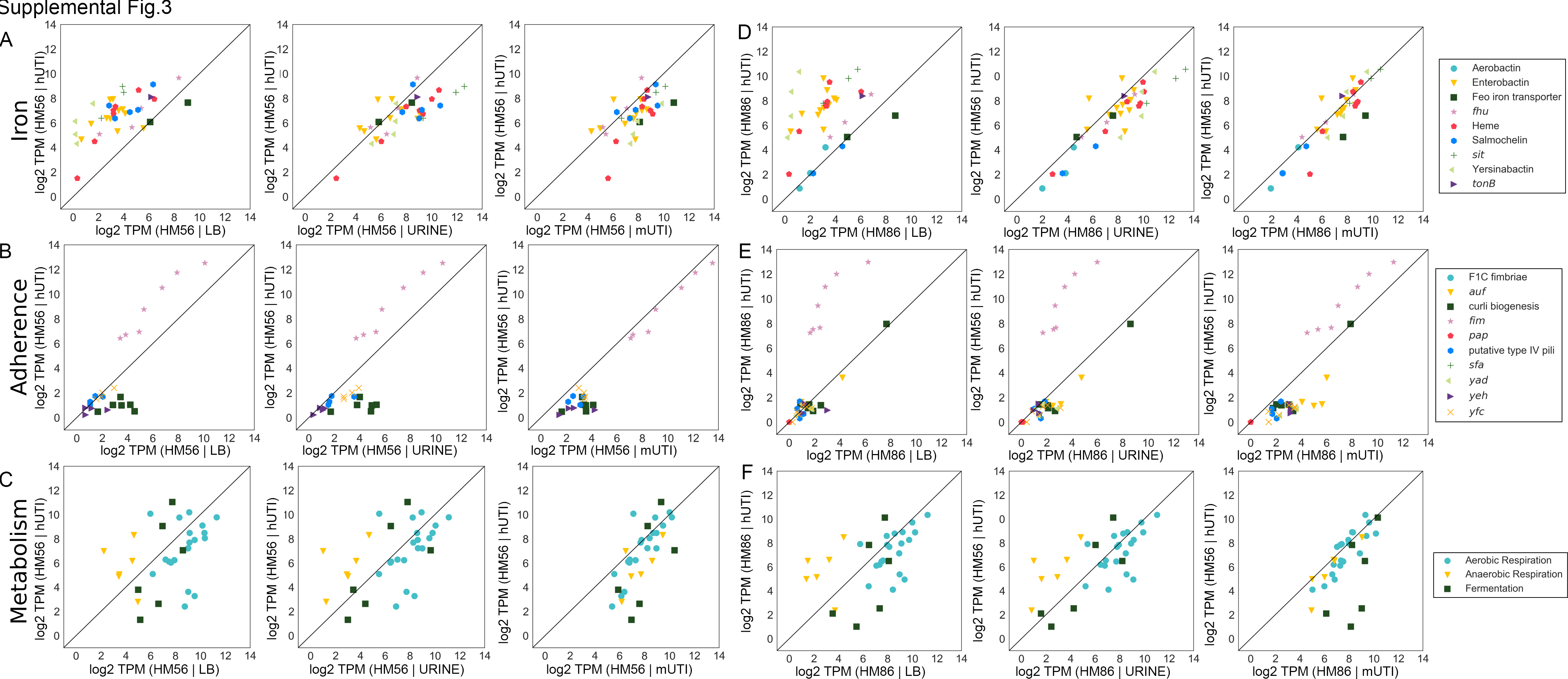

Supplement: FIG S3 [file mBio.01412-20-sf003.tif]
